# Multifaceted profiling of virus-specific CD8 T cells reveals distinct immune signatures against cytomegalovirus infection states during pregnancy

**DOI:** 10.1016/j.isci.2025.112416

**Published:** 2025-04-11

**Authors:** Ayumi Taguchi, Fumi Misumi, Shunsuke Teraguchi, Takeshi Nagamatsu, Shuhei Sakakibara, Tomohiro Otani, Mari Ichinose, David Priest, Kazuki Nakajima, Junko Nakamura, Ryoko Sawada, Tatsuo Suzutani, Toshiyuki Ikeda, Yutaka Nagura, Takayuki Iriyama, Daisuke Okuzaki, Hitoshi Okazaki, James B. Wing, Yasushi Hirota, Yutaka Osuga

**Affiliations:** 1 Laboratory of Human Single Cell Immunology, WPI Immunology Frontier Research Center, The University of Osaka, Osaka 565-0871, Japan; 2 Department of Obstetrics and Gynecology, Graduate School of Medicine, The University of Tokyo, Tokyo 113-8654, Japan; 3 Faculty of Data Science, Shiga University, Shiga 522-8522, Japan; 4 Department of Genome Informatics, Research Institute for Microbial Diseases, The University of Osaka, Osaka 565-0871, Japan; 5 Department of Obstetrics and Gynecology, International University of Health and Welfare, Narita 286-8686, Japan; 6 Laboratory of Systems Immunology, WPI Immunology Frontier Research Center, The University of Osaka, Osaka 565-0871, Japan; 7 Graduate School of Medical Safety Management, Jikei University of Health Care Sciences, Osaka 532-0003, Japan; 8 Department of Transfusion Medicine, The University of Tokyo, Tokyo 113-8655, Japan; 9 Laboratory Sciences, Department of Health Sciences, School of Health and Social Service, Saitama Prefectural University, Saitama 343-0036, Japan; 10 Department of Microbiology, Fukushima Medical University School of Medicine, Fukushima 960-1295, Japan; 11 Laboratory of Human Immunology (Single Cell Genomics), WPI Immunology Frontier Research Center, The University of Osaka, Osaka 565-0871, Japan; 12 Human Single Cell Immunology Team, Center for Infectious Disease Education and Research, The University of Osaka, Osaka 565-0871, Japan

**Keywords:** biological sciences, immunology, pregnancy

## Abstract

Anti-cytomegalovirus (CMV) serological testing, including the IgG avidity index (AI), is used to assess CMV infection phases during pregnancy. However, little is known about anti-CMV cellular immunity during pregnancy, particularly its relation to serological diagnosis. Herein, using MHC-dextramer single-cell RNA sequencing and flow cytometry, we characterized IE1 and pp65 CMV-antigen specific CD8 T cells from pregnant women with different anti-CMV serological patterns, including IgG^+^IgM^+^/AI-low, IgG^+^IgM^+^/AI-high, and IgG^+^IgM^−^. In IgG^+^IgM^+^/AI-low and IgG^+^IgM^+^/AI-high specimens, CMV-specific T cells consisted largely of effectors, with a minor but characteristic proportion of memory T cells, including HLA-DR-positive memory precursors and granzyme K-high memory cells reactive to IE1. Conversely, IgG^+^IgM^−^ cases had a distinctive expansion of pp65-specific terminally differentiated T effector memory with a signature of convergent clonal selection. Our findings revealed that different CMV infection phases have characteristic patterns of CD8 cell phenotype and antigen recognition, potentially offering a new approach for assessing congenital infection risk.

## Highlights

• CMV-specific T cells in pregnant women were characterized by MHC-dextramer scRNA-seq
• CMV-specific T cell profiles linked to antiviral antibody patterns were identified
• IgG^+^IgM^+^/AI-low donors exhibit a high proportion of IE1-specific Teffs
• IgG^+^IgM^−^ donors exhibit clonal convergence and expansion of pp65-specific Tmems

## Graphical abstract

**Figure:**
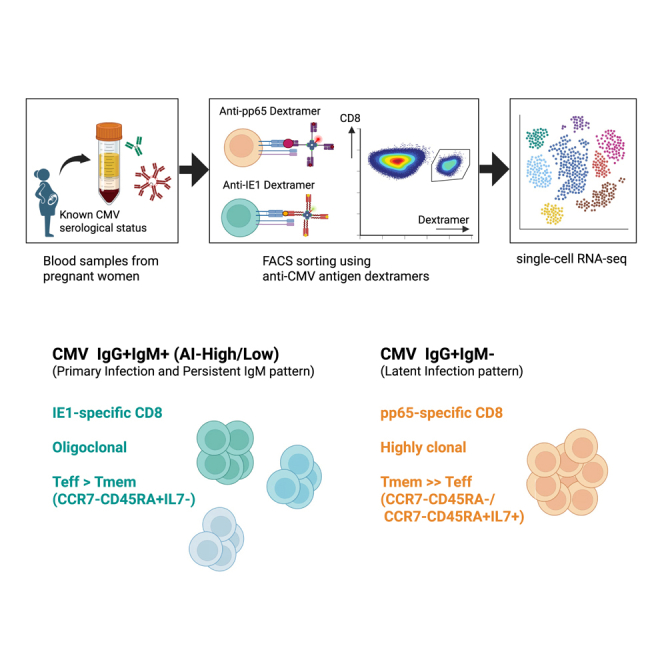

## Introduction

Cytomegalovirus (CMV) is a prevalent pathogen with a seroprevalence of 45%–100%, depending on region.^1^ In people with a healthy immune system, primary CMV infection is generally asymptomatic and shifts to a dormant state, which may reactivate sporadically later in life. However, in immunocompromised individuals, such as those with HIV/AIDS, organ transplant recipients, and neonates, CMV infection can cause serious symptoms.^2^^,^^3^^,^^4^

Congenital CMV infection (cCMV), caused by transmission from mother to fetus through the placenta, is a leading cause of sensorineural hearing loss and developmental disorders.^5^^,^^6^^,^^7^^,^^8^ Neonates born to mothers with primary infection during pregnancy are at a high risk for cCMV, with an incidence rate of 40%.^9^ cCMV infection also results from viral reactivation or reinfection with a different viral strain. However, the frequency of cCMV among seropositive mothers is limited compared to those with primary infection.^10^ Given this context, an accurate assessment of CMV infection phases during pregnancy is urgently needed.

CMV infection phases are typically diagnosed based on CMV serological status. The typical CMV latent infection phase is represented by CMV IgG positivity and IgM negativity. Theoretically, primary infection is diagnosed when anti-CMV seroconversion from negative to positive anti-CMV IgG is detected. However, such seroconversion is rarely confirmed because periodic checks for anti-CMV serostatus are not common in practice. Another issue is the difficulty in diagnosing primary infection based on anti-CMV IgM status. Similar to other viral infections, CMV IgM is detected within a couple of weeks of the primary infection, prior to the detection of CMV IgG.^11^ While most individuals become CMV IgM negative within a few months of the primary infection, some individuals exhibit CMV IgM positivity for an extended period. This phenomenon, called “persistent IgM,” makes the timing of primary infection difficult to estimate.^10^^,^^12^ Owing to the potential presence of persistent IgM, CMV IgG avidity index (AI) has been employed alongside CMV IgG and IgM testing to estimate the timing of primary CMV infection. This assay relies on the principle of antibody affinity maturation after infection. Consequently, the presence of IgM antibodies with low IgG avidity indicates a recent primary infection. However, the IgG avidity test has several limitations, and assays for IgG avidity have not been standardized.^10^^,^^13^^,^^14^ Thus, anti-CMV immune responses other than antibody titers should be investigated, and methods to accurately assess infectious phases should be explored.

Cytotoxic T cells play a pivotal role in protective immunity against viral infections. The characterization of CMV-specific CD8 T cells could facilitate a better understanding of the immune defense to control CMV activity during pregnancy and contribute to the improved diagnosis of cCMV. Previous studies on CMV-specific CD8 T cells primarily focused on those during latent CMV infection in healthy individuals or reactivation after stem cell transplantation.^15^^,^^16^ However, the functional profiles of CMV-specific CD8 T cells in pregnant women shortly after primary infection and in those with persistent IgM remain elusive.

In this study, we aimed to characterize the transcriptional diversity, clonal composition, and viral target antigens of CMV-specific CD8 T cells among pregnant women with different anti-CMV serological patterns, including IgG^+^IgM^+^/AI-low, IgG^+^IgM^+^/AI-high, and IgG^+^IgM^−^. Single-cell RNA sequencing (scRNA-seq) combined with major histocompatibility complex (MHC)-peptide dextramers revealed that CMV immediate-early 1 (IE1)-specific short-lived effectors and proliferating cells were characteristic of the anti-CMV IgG^+^IgM^+^/AI-low or IgG^+^IgM^+^/AI-high cases. By contrast, clonal expansion of phosphoprotein 65 (pp65)-specific terminal effector memory was prominent in the CMV-specific CD8 T cells from the IgG^+^IgM^−^ cases. Our findings provide insights into CD8 T cell response to different phases of CMV infection, potentially allowing the accurate assessment of cCMV risk during pregnancy.

## Results

### Assessment of serological diagnosis for congenital CMV risk

**Figure 1:**
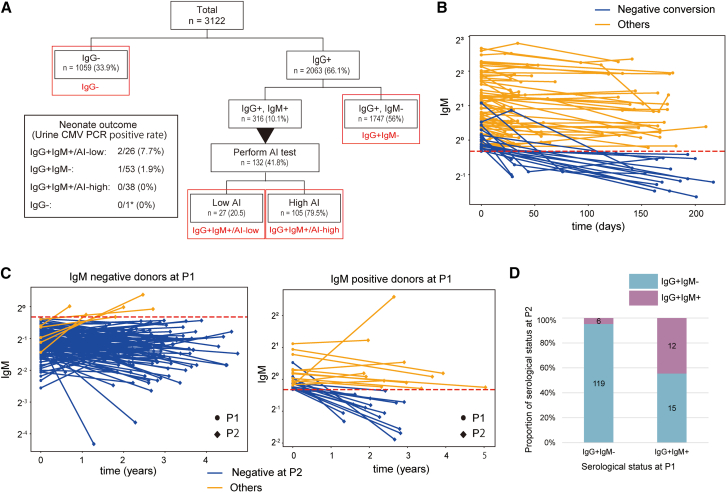
Kinetics of CMV IgM decay vary widely among individuals (A) Summary of anti-CMV antibody status in pregnant donors and their neonatal outcomes. Results from CMV serological tests using enzyme immunoassay (EIA) in early pregnancy (<16 weeks) and their corresponding neonatal outcome are summarized (*N* = 3122). Donors were classified into the following four groups: IgG^+^IgM^+^/AI-low group, CMV IgG-positive and IgM-positive with low IgG avidity index (AI); IgG^+^IgM^+^/AI-high group, IgG-positive and IgM-positive with high AI; IgG^+^IgM^−^ group, CMV IgG-positive and CMV IgM-negative; and IgG^−^ group, CMV IgG-negative. Asterisk indicates a case in which serological data were converted to IgG and IgM positive in the late pregnancy. (B) Temporal changes in IgM titers among donors who tested CMV IgG^+^ and IgM^+^ within 16 weeks of conception. Donors who had multiple test results during pregnancy were included (*N* = 95). The threshold of positivity was set at IgM titer 0.8. “Day 0” refers to the date of the first serological testing, and time (days) indicates time after first serological testing. Cases with negative conversion are marked in blue, and those without negative conversion are marked in orange. (C) Changes in CMV IgM titers between two consecutive pregnancies. Left, IgG-positive, IgM-negative (IgG+ IgM-) donors (*N* = 125); right, IgG-positive, IgM-positive (IgG+ IgM+) donors (*N* = 27) in their first pregnancy in the cohort (P1). The measurements at the time of first pregnancy (P1) are marked with ●, and those at the time of second pregnancy (P2) are marked with ♦. “Time” indicates the time (year) from the date of the initial examination at first pregnancy (P1) to the time of the initial examination at second pregnancy (P2). Cases with positive IgM at second pregnancy are marked in orange, and cases with negative IgM are marked in blue. (D) Summary of serological tests in the second pregnancy (P2) based on serological status in the first pregnancy (P1).

To evaluate the current serological diagnoses in Japan, we retrospectively analyzed the anti-CMV antibody test results of pregnant female donors enrolled in a maternal antibody screening program at the University of Tokyo Hospital, Japan (Table S1). Based on CMV serological test results in early pregnancy (<16 weeks gestation), donors were stratified into four groups: CMV IgG negative (IgG^−^), CMV IgG-positive and IgM-negative (IgG^+^IgM^−^), CMV IgG- and IgM-positive and IgG AI-high (IgG^+^IgM^+^/AI-high), and CMV IgG- and IgM-positive and IgG AI-low (IgG^+^IgM^+^/AI-low) (Figure 1A). Among 3,122 pregnant women at 16 weeks of conception, 1,059 pregnant women (33.9%) were IgG^−^ and diagnosed as uninfected. After serological testing, these donors were provided with education to avoid CMV exposure during pregnancy. Upon follow up, 740 pregnant women underwent CMV serological testing in late pregnancy. Of these women, 735 were IgG negative, one was IgG negative and IgM positive, three were IgG positive and IgM negative, and one was IgG and IgM positive. Neonatal urine CMV PCR was performed on one IgG- and IgM-positive case, but no evidence of cCMV was observed in the neonate. Conversely, 1,747 (56.0%) tested positive for CMV IgG and negative for IgM (hereafter referred to as IgG^+^IgM^−^), indicating latent infection and a low risk of cCMV. In this group, 53 donors underwent neonatal urine PCR testing for CMV DNA, and one (1.9%) tested positive for cCMV. The remaining 316 patients in the main cohort (10.1%) tested positive for CMV IgG and IgM (hereafter referred to as IgG^+^IgM^+^) (Figure 1A). Among them, 132 IgG^+^IgM^+^ pregnant women whose IgM titers were higher than 1.2 were further tested for CMV AI. Results showed that 27 (20.5%) had a low AI (hereafter referred to as IgG^+^IgM^+^/AI-low), indicating a recent primary CMV infection and relatively high cCMV risk (Figure 1A). Among these IgG+IgM+/AI-low donors, CMV DNA was detected in neonatal urine by PCR in 7.7% (2/26) of those tested. By contrast, among the IgG^+^ IgM^+^ donors, 105 (79.5%) showed high AI (hereafter referred to as IgG^+^IgM^+^/AI-high), and no cases of cCMV were found among the 38 neonatal urine PCR-tested donors in this group (Figure 1A). Thus, the occurrence of cCMV was higher in pregnant women with IgG+IgM+/AI-low than in those with IgG+IgM+/AI-high. This result was consistent with the current serological diagnosis for cCMV risk using CMV AI.

We next investigated the temporal changes in IgM titers among donors who tested CMV IgG^+^ and IgM^+^ within 16 weeks of conception. Of the 316 IgG and IgM-seropositive donors in our cohort, 95 had multiple IgM results. Examination of transition in IgM titers over time showed a diverse pattern, with some titers decreasing and others remaining high (Figure 1B). Among them, 50 had multiple test results with an interval of more than 100 days between the initial and subsequent tests or confirmed negative conversion of IgM during pregnancy. Overall, the CMV IgM titers in these donors declined during pregnancy, although to varying degrees: 43 of the 50 donors showed a decrease in CMV IgM titers (Figure S1). Of these, 19 underwent seroconversion to IgM-negative status. However, 31 (seven increased and 24 decreased) of the 50 donors remained positive for CMV IgM over ≥100 days (Figure S1).

We further examined serological changes in 240 donors with two consecutive pregnancies during the CMV antibody screening program (Table S2). Among the 152 donors who tested positive for CMV IgG at their first pregnancy (P1), 27 tested CMV IgG^+^IgM^+^ within 16 weeks of P1. Among these donors, 12 (44.4%) remained positive for CMV IgM during their subsequent pregnancy (P2) (Figures 1C and 1D). Conversely, only six (4.8%) of the 125 pregnant women who were IgG^+^IgM^−^ at P1 became IgG^+^IgM^+^ at P2 (Figures 1C and 1D). This conversion may have been due to viral reactivation or reinfection. From these results, a significant proportion of IgG^+^IgM^+^ donors exhibited the IgG^+^IgM^+^ pattern in their consecutive pregnancies, representing the abnormal immune response for CMV control in these donors.

### Functional diversity in the CD8 T cell response against CMV infection in pregnant donors

These results underscore the difficulty of interpreting CMV serological patterns, particularly in pregnant women with CMV IgG^+^IgM^+^. Furthermore, this raises the question of how the CMV serological status corresponds to the anti-CMV response, especially in terms of cellular immunity. To gain insights into cellular immunity, we analyzed CMV-specific CD8 T cells at the single-cell level using MHC-dextramer scRNA-seq. We selected HLA-A∗24:02-positive donors for this analysis because this is the most common HLA-A type in the Japanese and other East Asian populations.^17^ CD8 T cells specific to one of the three immunodominant viral epitopes (IE1-AYAQKIFKI, pp65-QYDPVAALF, and pp65-VYALPLKML) were sorted from peripheral blood mononuclear cells (PBMCs) of HLA-A∗24:02 pregnant women from donors with different CMV serological patterns (15 time points from 10 donors: *n* = 15, *N* = 10) (Table 1) and subjected to droplet-based scRNA-seq combined with T cell receptor (TCR) repertoire sequencing (Figure S2). In certain donors (donor ID: XA0086, XA0116, and XA0119) initially exhibiting an IgG^+^IgM^+^/AI-low serological pattern, their CMV AI surpassed the threshold after the middle of pregnancy. Consequently, the specimens from these donors at later time points were classified into the IgG^+^IgM^+^/AI-high group (Table 1). Time course analysis was also performed for these donors to assess changes in CMV-specific CD8 T cell profiles by AI. Gestational weeks at sample collection are summarized in Table 1. We confirmed the specific isolation of CMV-specific T cells using gating settings, where no dextramer-positive cells were observed in the CMV-seronegative samples (Figure S3). The frequencies of dextramer-positive cells varied among individual specimens, ranging from 0.2% to 16.4% of CD8 T cells (Figure S4). Deciphering barcodes on the dextramer reagents identified T cells captured by HLA-A∗24:02-IE1-AYAQKIFKI and HLA-A∗24:02-pp65-QYDPVAALF. However, we barely isolated pp65-VYALPLKML-specific T cells with less than four cells per sample and a total of 10 cells. Therefore, we excluded these cells from subsequent analyses. The number of cells detected in the scRNA-seq data for each donor is summarized in Table S3. Of the 9,056 cells used in the analysis, 3,402 (37.5%) and 52 (0.6%) were from XA0045 and XA0104, respectively, which had the highest and lowest percentages of CMV dextramer-positive cells, respectively. Based on the scRNA-seq data, the median expression of CD8 T cells in each sample was reflected in the uniform manifold approximation and projection (UMAP) (Figure S5). The profiles of CMV-specific CD8 T cells from donors belonging to the different serological categories showed minimal changes over time.

**Table 1:** Clinical summary of pregnant donors for single-cell RNA sequence

Donor ID	Age	Gravida	Parity	Gestational age (weeks)	IgG	IgM	IgG Avidity	Group	Sample ID	Neonate CMV DNA
XA0045	38	2	1	13	9.3	6.33	NE			Negative
				17	17	5.71	0.77			
				21	17.5	2.89	0.922	IgG^+^IgM^+^/AI-high	M1-1	
				29	18	4.53	0.914	IgG^+^IgM^+^/AI-high		
				36	13.7	1.78	0.91	IgG^+^IgM^+^/AI-high	M1-2	
XA0068	33	2	1	9	2	0.92	NE			Negative
				13	24.1	0.59	0.92	IgG^+^IgM^-^	L1-1	
				30	NE	NE	0.908	IgG^+^IgM^-^		
				36	18.8	0.44	0.888	IgG^+^IgM^-^	L1-2	
XA0086	28	1	0	16	3.3	6.3	NE			Negative
				18	7.2	8.03	−0.01	IgG^+^IgM^+^/AI-low		
				21	27	5.16	0.022	IgG^+^IgM^+^/AI-low	P1-1	
				26	30.5	3.5	0.084	IgG^+^IgM^+^/AI-low		
				36	NE	NE	0.574	IgG^+^IgM^+^/AI-high	P1-2	
XB0031	35	2	0	20	2	0.5	NE	IgG^-^	N1	Negative
XB0001	34	2	1	12	13.2	0.31	NE	IgG^+^IgM^-^	L2	Negative
XA0116	32	5	2	12	8.3	2.33	NE			Positive
				13	7.3	2.12	0.408	IgG^+^IgM^+^/AI-low		
				19	5.1	1.4	0.545	IgG^+^IgM^+^/AI-high	P2-1	
				28	6.3	0.96	0.689	IgG^+^IgM^+^/AI-high	P2-2	
XA0119	33	2	1	9	NE	19.2	NE			Positive^$^
				11	11.4	3.64	0.077	IgG^+^IgM^+^/AI-low	P3-1	
				20	NE	NE	0.732	IgG^+^IgM^+^/AI-high	P3-2	
XA0104	26	1	0	16	10.5	3.42	NE			Negative
				23	23.5	3.18	0.221	IgG^+^IgM^+^/AI-low	P4	
				30	13.5	3.76	0.286	IgG^+^IgM^+^/AI-low		
				35	NE	NE	0.286	IgG^+^IgM^+^/AI-low		
XA0109	36	2	1	12	6.9	1.43	NE			Negative
				18	13.1	1.47	0.796	IgG^+^IgM^+^/AI-high	M2	
XA0108	32	3	2	10	11.5	2.32	NE			Negative
				15	14.7	2.25	0.882	IgG^+^IgM^+^/AI-high	M3	

All serological data of donors who performed MHC-dextramer scRNA-seq are listed. NE, not evaluated; $, Amniotic fluid.

**Figure 2:**
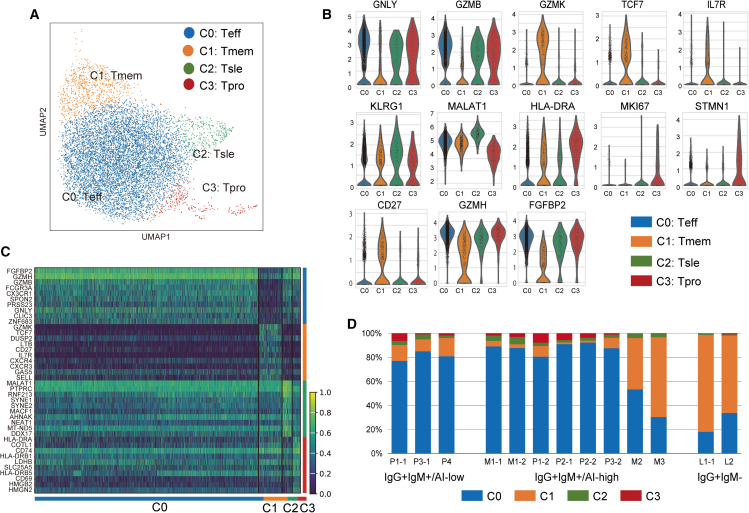
scRNA-seq analysis of CMV-specific T cells in pregnant women (A) UMAP projections of the single cell transcriptome of CMV-specific CD8 T cells captured by HLA-A∗24:02 AYAQKIFKI (IE1) or HLA-A∗24:02 QYDPVAALF (pp65) MHC-dextramers. CMV-specific CD8 T cells were divided into four clusters: C0, Teff; C1, Tmem; C2, Tsle; and C3, Tpro. (B) Violin plots showing the expression level of characteristic marker genes in each cluster. Y axis is presented on a log scale. (C) Heatmap representation of DEGs in each cluster. The top 10 DEGs in each cluster are shown. (D) Proportion of T cell subsets in individual samples according to serological groups.

We subsequently annotated the single-cell transcriptome data and identified four distinct clusters (C0, C1, C2, and C3) of CMV-specific CD8 T cells (Figure 2A). C0 represented CD8 T effectors (Teff), as they were rich in effector genes such as *FGFBP2*, *GZMH*, *GZMB*, and *GNLY* (Figures 2B and 2C). C1 was a memory T subset (Tmem) marked by increased expression of *GZMK*, *TCF7*, *CD27*, and *IL7R* (Figures 2B and 2C). C2 was equivalent to the previously reported KLRG1^+^ IL7R^−^ short-lived effector (Tsle)^18^ and was further marked by the high expression of *MALAT1*, a long noncoding RNA that induces terminal differentiation.^19^ Consistently, they also showed increased expression of several mitochondrial genes in dying cells. C3 included proliferating T cells (Tpro), as they highly expressed *HLA-DRA* and genes related to cell cycle progression (*STMN1* and *MKI67*) (Figures 2B and 2C). Collectively, MHC-dextramer scRNA-seq results indicated functional diversity in CMV-specific CD8 T cells in pregnant women.

A comparison of the distribution of CD8 T cells among the groups revealed distinctive distribution patterns of CMV-specific T cells among these clusters between the IgG^+^IgM^−^ group and the IgM-positive groups (Figures 2D and S6). Tmems dominated over Teffs in the distribution of CMV-specific T cells in the IgG^+^IgM^−^ group (Figure 2D). By contrast, the IgG^+^IgM^+^/AI-low group was characterized by a large fraction of Teff (C0) and a small fraction of Tmem (C1). Meanwhile, the IgG^+^IgM^+^/AI-high group comprised a mixture of specimens with different subset distribution patterns. Six of the eight samples (M1-1, M1-2, P1-2, P2-1, P2-2, and P3-2) exhibited subset compositions similar to those of the IgG^+^IgM^+^/AI-low group, whereas two samples (M2 and M3) contained higher proportions of Tmems and lower proportions of Teffs than those contained by the six other samples (Figure 2D).

### Serological group-dependent and independent features unveiled by CMV-specific memory T cell classification

**Figure 3:**
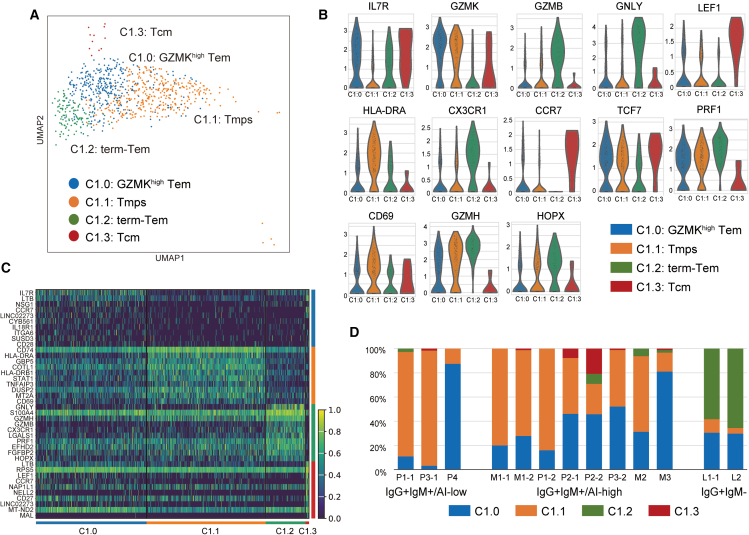
Functional compositions of CMV-specific memory by serological groups (A) UMAP projection of the single cell transcriptome of the CMV-specific memory fraction. CD8 T cells in C1 (Tmem) were divided into four clusters: C1.0, GZMK^high^ Tem; C1.1, Tmp; C1.2 term-Tem; C1.3, Tcm. (B) Violin plots showing the expression levels of characteristic marker genes in each cluster. Y axis is presented on a log scale. (C) Heatmap representation of DEGs in each cluster. The top 10 DEGs in each cluster are shown. (D) Proportion of memory T cell subsets in individual samples according to serological groups.

Memory T cell responses are crucial for immunological surveillance against viral reactivation and reinfection. To understand the phenotypic differences in antiviral memory T cells during pregnancy with different viral infection states, we further classified the C1 subset (Tmem) into four subclusters (C1.0, C1.1, C1.2, and C1.3) (Figure 3A). C1.0 was characterized by high *GZMK* and low *GZMB* expression (GZMK^high^ Tem) (Figure 3B and 3C). C1.1 corresponded to previously reported memory T precursors (Tmps) with high levels of *GZMK*, *HLA-DRA*, *CD69*, and *TCF7* and low levels of *GZMB* and *IL7R*^20^ (Figures 3B and 3C). C1.2 exhibited a distinct effector memory T phenotype, characterized by higher expression of multiple cytotoxic granules and effector markers (*GZMB*, *GNLY, GZMH*, *PRF1*, and *CX3CR1*) and lower expression of *GZMK* and *TCF7* than those shown by other memory subclusters (Figures 3B and 3C). Cells in this subcluster also showed elevated expression of *HOPX*, a transcription cofactor crucial for the maintenance of T effector precursors^21^ (Figure 3C). These transcriptional features indicate that the cells in C1.2 were terminally differentiated Tem (term-Tem). C1.3, although scarce in absolute number, represented central memory T cells (Tcm) with high expression of *CCR7* and *LEF* (Figures 3B and 3C).

We aimed to identify serological group-specific characteristics in the composition of memory T cell subclusters. The expansion of term-Tems (C1.2) was distinctive for the IgG^+^IgM^−^ group, constituting 60%–70% of the memory fraction (Figure 3D). By contrast, the specimens in the IgG^+^IgM^+^/AI-low and IgG^+^IgM^+^/AI-high groups exhibited an expansion of memory subclusters with high *GZMK* expression, namely, GZMK^high^-Tem (C1.0) and Tmp (C1.1) but with different compositions (Figure 3B). P1-1 and P3-1 in the IgG^+^IgM^+^/AI-low group and M1-1, M1-2, P3-2, and M2 in the IgG^+^IgM^+^/AI-high group had >50% of Tmps in the memory fraction (Figure 3B), suggesting early memory formation in these donors. P2-1 and P2-2 contained substantial proportions of Tcm (C1.3) (Figure 3B). Overall, the expansion of term-Tem was a distinctive feature of the IgG^+^IgM^−^ group, whereas CMV-specific memory T cells exhibited a relatively heterogeneous subcluster composition in the IgG^+^IgM^+^/AI-low and IgG^+^IgM^+^/AI-high groups.

The profiles of CMV-specific CD8 T cells were then compared based on the presence or absence of cCMV infection. However, no obvious differences in the distribution on the UMAP, CD8 T cell subtypes, or memory CD8 T cell subtypes were found (Figure S7).

### Targets, repertoire, and clonal compositions of CMV-specific T cells

**Figure 4:**
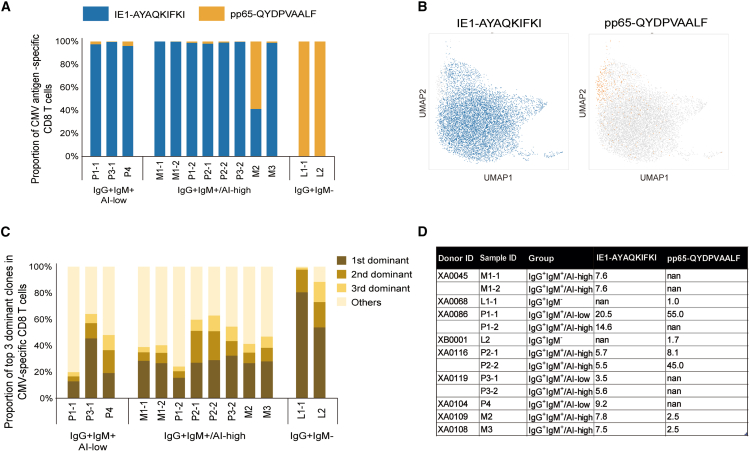
Target antigen and clonality of CMV-specific CD8 T cells (A) Bar graph showing the proportion of IE1-and pp65-specific CD8 T cells determined by MHC-dextramers in individual samples according to serological groups. (B) UMAP projections of the single cell transcriptome of the IE1-AYAQKIFKI-, or pp65-QYDPAVAALF-specific CD8 T cells. (C) Proportions of the top 3 largest clones in CMV-specific T cells. (D) Inverse Simpson index (ISI) values of pp65-and IE-specific clones.

We further examined the target epitope preference and clonal composition of virus-specific T cells in individual donors. The CMV-specific T cells in the IgG^+^IgM^+^/AI-low and IgG^+^IgM^+^/AI-high groups, except for specimen M2, preferentially targeted IE1 (Figure 4A), whereas those in the IgG^+^IgM^−^ group exclusively targeted pp65 (Figure 4A). Notably, the IE1-specific clones were distributed into diverse subsets of Tem, Teff, Tsle, and Tpro (Figure 4B), suggesting an early T cell response in these specimens. By contrast, the clonotypes of pp65-specific clones were predominantly polarized toward term-Tems (Figure 4B). Moreover, the CMV-specific T cells in the IgG^+^IgM^−^ group showed extremely high clonality; the top three largest clonotypes accounted for 98.5% and 88.5% of the CMV-specific CD8 T cells in L1-1 and L2, respectively (Figure 4C). The clonal composition of CMV-specific T cells in the IgG^+^IgM^+^/AI-low and IgG^+^IgM^+^/AI-high groups was somewhat diverse compared to that in the IgG^+^IgM^−^ group, but the top three largest clonotypes still accounted for approximately 20%–60% of the CMV-specific T cells (Figure 4C). The diversity of TCR repertoire was estimated using the inverse Simpson’s index (ISI), in which a greater score indicates higher diversity.^22^^,^^23^ The ISI values of the pp65-specific clones in L1-1 and L2 from the IgG^+^IgM^−^ group were 1.00 and 1.7, respectively, whereas those of the IE1-specific clones in the IgG^+^IgM^+^/AI-low and IgG^+^IgM^+^/AI-high groups varied from 3.5 to 20.5 (Figure 4D). Collectively, the extremely high clonality of pp65-specific Tems was a hallmark of the IgG^+^IgM^−^ group, whereas oligoclonal IE-1-specific T cells exhibiting functional heterogeneity characterized the CMV-specific T cells from donors in the IgG^+^IgM^+^/AI-low and IgG^+^IgM^+^/AI-high groups.

**Figure 5:**
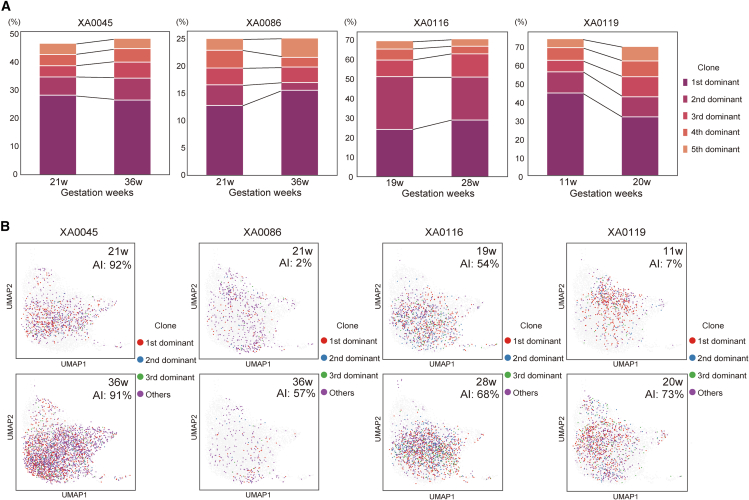
Longitudinal changes in CMV-specific clones (A) Bar graphs showing the longitudinal changes in the percentages of the five largest clones for donors XA0045, XA0086, XA0116, and XA0119. (B) UMAP projections illustrating the longitudinal changes in the single cell transcriptome of CMV-specific clones in these donors. AI, IgG avidity index.

We subsequently conducted a longitudinal analysis. The proportions and cluster distribution of large clones did not change dramatically between the two blood collections at intervals >60 days during pregnancy (Figures 5A and 5B and Table S4). In particular, two cases (XA0086 [P1-1 and P1-2] and XA0119 [P3-1 and P3-2]) showed an increase in AI at the second time point. However, even in these two cases, CMV-specific T cell characteristics were maintained throughout the gestational period and did not change based on AI, suggesting that changes in AI did not sensitively alter T cell profiles.

**Figure 6:**
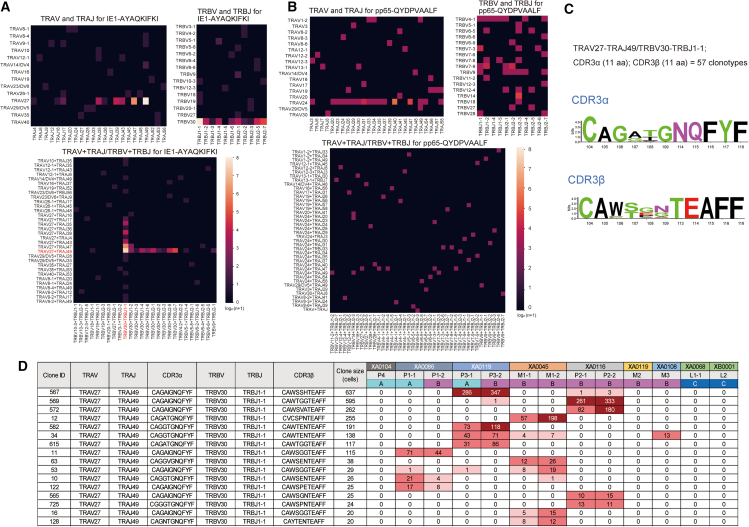
TCR repertoire analysis of CMV-specific T cells (A and B) TCR Vα-Jα, and Vβ-Jβ gene segment usage and Vα-Jα, Vβ -Jβ pairings in IE1-specific T cell clonotypes (A) and pp65-specific T cell clonotypes (B). In the visualization, the contribution of each QYDPVAALF clone was normalized by multiplying #AYA/#QYD to ensures that the same color represents the same fraction across respective T cell population. #AYA: total number of IE1-AYAQKIFKI clones, #QYD: total number of pp65-QYDPAVAALF clones. (C and D) Examples of similar T clonotypes specific to IE1 across donors. Sequencing logos of CDR3α and β from all clonotypes encoded by TRAV27-TRAJ19 and TRBV30-TRBJ1-1 with 11 amino acid-long CDR3α and β in the dataset (C). Clonal distribution across donors of the top 20 clonotypes in this class (D). Group A, IgG^+^IgM^+^/AI-low; group B, IgG^+^IgM^+^/AI-high; and group C, IgG^+^IgM^−^.

A repertoire analysis of 451 clonotypes composed of 7,773 cells, in which a single pair of TCR chains was detected, highlighted the exclusive use of *TRAV27* (94.1%) and *TRBV30* (95.6%) in IE-1-specific clonotypes (Figure 6A and Table S5). In contrast, pp65-specific clonotypes were encoded by a wide variety of TRAV and TRBV segments: *TRAV24* was the most prevalent segment, accounting for 53.3% (24/45) (Figures 6B and Table S5). Further analysis revealed that highly similar clonotypes encoded by *TRAV27*-*TRAJ49* and *TRBV30-TRBJ1-1* were induced in nine specimens from the IgG^+^IgM^+^/AI-low and IgG^+^IgM^+^/AI-high groups, comprising five donors (Table S6). For instance, 57 clonotypes encoded by *TRAV27*-*TRAJ49* and *TRBV30-TRBJ1-1* with 11-aa CDR3α/β with high similarity were found in the IE1-specific repertoire of the IgG^+^IgM^+^/AI-low and IgG^+^IgM^+^/AI-high groups (Figure 6C and Table S6). However, we did not find a similar enrichment for public clones in the pp65-specific repertoire. Clonotypes with ≥20 cells in this class are shown in Figure 6D. Overall, a limited TCR repertoire elicited IE1-specific T cell responses in most specimens from the IgG^+^IgM^+^/AI-low and IgG^+^IgM^+^/AI-high groups. The diverse clonality of IE1-specific T cells, as indicated by the ISI, consisted of similar TCR clonotypes.

### Surface phenotypes of CMV-specific CD8 T cells in pregnant donors

**Figure 7:**
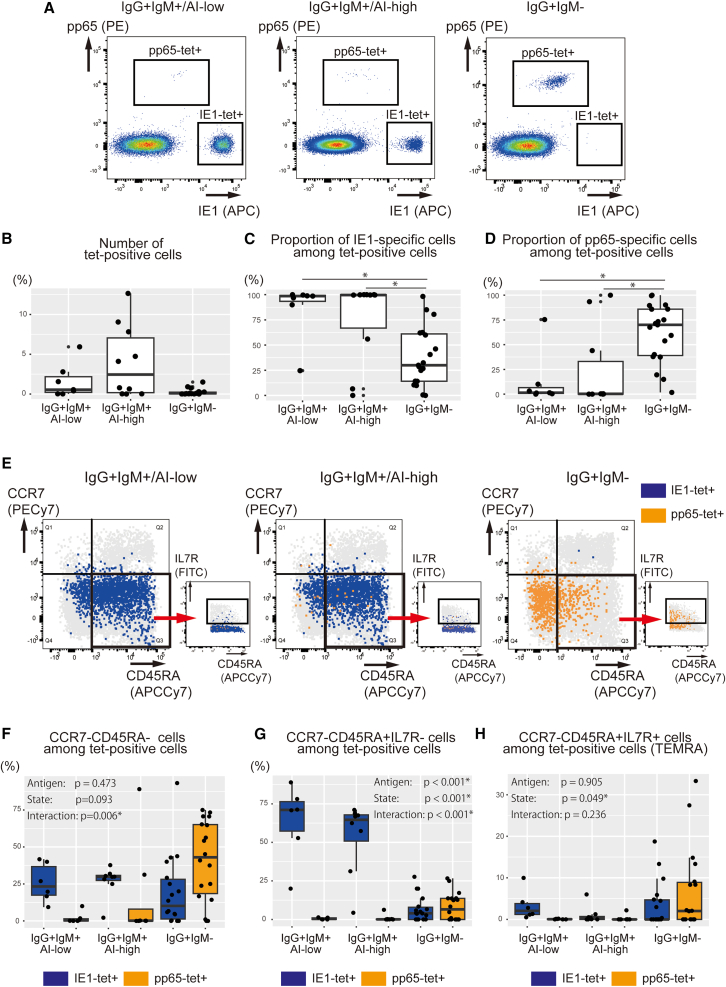
Surface phenotypes of CMV-specific CD8 T cells differ by target antigens and infection states (A) Detection of CMV-specific CD8 T cells by flow cytometry. Representative FACS plots gated on 7AAD^−^ CD8 cells stained with APC-conjugated HLA-A∗24:02 AYAQKIFKI (IE1) and PE-conjugated HLA-A∗24:02 QYDPVAALF (pp65) tetramers: left panel, IgG^+^IgM^+^/AI-low group; middle panel, IgG^+^IgM^+^/AI-high group; and right panel, IgG^+^IgM^−^ group. (B) Number of tetramer-positive cells by CMV serological status. The total number of HLA-A∗24:02 IE1-AYAQKIFKI-specific and pp65-QYDPAVAALF-specific cells was represented as tetramer-positive cells. (C) Proportion of IE1-AYAQKIFKI-specific cells among tetramer-positive cells. (D) Proportion of pp65-QYDPAVAALF-specific cells among tetramer-positive cells. (E–H) Surface phenotypes of CMV-specific CD8 T cells at different phases of infection. Representative FACS plots gated on 7AAD^−^ CD8 cells: left panel, IgG^+^IgM^+^/AI-low group; middle panel, IgG^+^IgM^+^/AI-high group; and right panel, IgG^+^IgM^−^ group (E). Proportion of CD45RA^−^ CCR7^-^ effector memory T cells (F), proportion of CD45RA^+^ CCR7^-^ IL7R^−^ effector T cells (G), and proportion of CD45RA^+^ CCR7^-^ IL7R^+^ TEMRA cells (H) among tetramer-positive CD8 T cells. The Kruskal-Wallis test with Holm correction was used in (B–D), and a two-way ANOVA was used in (F–H). The boxes represent the interquartile range (IQR) of the data, with the horizontal line inside each box indicating the median and whiskers extend to the most extreme data points within 1.5 times the IQR from Q1 and Q3 (B–D, G − H). Antigen: Whether the antigen (IE1 or pp65) affected the change in the proportion of each fraction; State: Whether infectious status (IgG^+^IgM^+^/AI-low, IgG^+^IgM^+^/AI-high, or IgG^+^IgM^−^) affected the change in the percentage of each fraction; Interaction: Whether “antigen” and “state” interact with each other in the proportion of each fraction (F–H). Asterisks indicate statistical significance (*p* < 0.05). ng tetramer-positive cells.

Flow cytometry was performed to examine whether the distinct profiles of virus-specific T cells could also be determined using surface markers. We analyzed 36 samples (IgG^+^IgM^+^/AI-low group: *n* = 7, IgG^+^IgM^+^/AI-high group: *n* = 10, and IgG^+^IgM^−^ group: *n* = 19) in which more than 5,000 CD8 T cells were obtained. The MHC-peptide tetramers identified CMV-specific T cells in a range of 0.007%–12.6% (IgG^+^IgM^+^/AI-low group: 0.018%–5.93%, IgG^+^IgM^+^/AI-high group: 0.007%–12.6%, and IgG^+^IgM^−^ group: 0.007%–1.50%) among CD8 T cells from a different cohort obtained at the same hospital (Figures 7A and 7B). These results were consistent with the findings of MHC-dextramer scRNA-seq, confirming that the vast majority of virus-specific T cells targeted IE1 in the IgG^+^IgM^+^/AI-low and IgG^+^IgM^+^/AI-high groups, whereas pp65-specific T cells were dominant over IE1-specific cells in the IgG^+^IgM^−^ group (Figures 7C and 7D).

The CMV-specific T cells in the IgG^+^IgM^+^/AI-low and IgG^+^IgM^+^/AI-high groups were restricted to IE1-specific cells, comprising a large fraction of CCR7^-^ CD45RA^+^ IL7R^−^ effectors and a small fraction of CCR7^-^ CD45RA^−^ effector memory T cells (Figures 7E, 7F, and 7G). By contrast, the virus-specific T cells in the IgG^+^IgM^−^ group were characterized by memory T cells consisting of CCR7^-^ CD45RA^−^ effector memory T cells and CCR7^-^ CD45RA^+^ IL7R^+^ terminally differentiated effector memory T cells (TEMRA) (Figures 7F and 7H). In agreement with MHC-dextramer scRNA-seq, tetramer staining and cell surface marker analysis revealed polarized differentiation of IE1-specific T cells into effectors in the IgG^+^IgM^+^/AI-low and IgG^+^IgM^+^/AI-high groups and viral-specific T cells into memory T cells in the IgG^+^IgM^−^ group.

## Discussion

In this study, we sought to clarify whether CMV-specific CD8 T cell responses are associated with anti-CMV serological patterns. Using MHC-dextramer scRNA-seq analysis, we investigated the effector/memory subset composition and variations in the functional properties of the memory subsets. We further assessed clonal characteristics, focusing on the IE1 and pp65 antigen recognition and clonal expansion of CMV-specific CD8 T cells.

The IgG^+^IgM^−^ population considered to have a history of infection before pregnancy showed distinct subset compositions of CMV-specific T cells in terms of memory cell properties and clone diversity when compared with the IgG^+^IgM^+^/AI-low and IgG^+^IgM^+^/AI-high populations. The CMV-specific T cells in the IgG^+^IgM^−^ group comprised large memory and small effector subsets with robust clonal expansion of pp65-specific T cells. This finding may reflect a unique CD8 T cell response to CMV, known as “memory inflation,”^24^ which is a persistent increase in specific T cell clones due to repetitive responses to specific antigens during latent infection. Making a clear contrast from the IgG^+^IgM^−^ group, the CMV-specific T cells in the population diagnosed as “primary infection” in the interpretation of serological phenotype (IgG^+^IgM^+^/AI-low) demonstrated exclusively targeted IE1 and displayed small memory and large effector subset distribution. The findings in the IgG^+^IgM^+^/AI-low and IgG^+^IgM^−^ groups suggest that the subset features of CMV-specific T cells align with the serology-based differential diagnosis of primary and latent (past) infection, which is commonly conducted in current obstetric practice. In the IgG^+^IgM^+^/AI-high group, in which the serological test results were difficult to interpret, the properties of CMV-specific CD8 T cells were heterogeneous in terms of the memory/effector functional fraction, antigen recognition, and clonal diversity.

Four subclusters with distinct mRNA expression patterns were identified in the phenotypic analysis of the CMV-specific CD8 T memory cell subset. The term-Tem subcluster, characterized by increased gene expression of multiple cytotoxic granules, including *GZMB* and *GZMH*, was dominant in the population with latent infection (IgG^+^IgM^−^ group). This finding suggests that sufficient time elapsed after CMV infection leads to the formation of functionally mature memory T cells. By contrast, in the IgG^+^IgM^+^/AI-low and IgG^+^IgM^+^/AI-high groups, CMV-specific memory T cells were a mixture of Tmps and GZMK^high^ Tems, functionally immature subsets. The induction of *GZMK*-expressing T effector memory cells has been observed in the initial stages of viral infections other than CMV, such as SARS-CoV-2 and influenza virus.^25^^,^^26^ The difference between the IgG^+^IgM^+^/AI-low and IgG^+^IgM^−^ groups can be ascribed to the difference in the duration following CMV infection. However, some IgM-positive conditions in the IgG^+^IgM^+^/AI-high group may have persisted for years; thus, other factors aside from the duration of infection may explain the difference. A recent study has proposed that viral antigen presentation by non-professional antigen-presenting cells, such as lymphatic endothelial cells, is crucial for the inflation and differentiation of terminally differentiated effector memory T cells in mice infected with murine cytomegalovirus (MCMV).^27^ In this context, the paucity of term-Tems in the IgG^+^IgM^+^/AI-low and IgG^+^IgM^−^ groups may be due to a lack of antigen presentation by a certain cell type during persistent infection, where CMV fails to reside in a cell type capable of serving as a latent viral reservoir.

Although diverse clones of IE1-specific CD8 T cells with varied memory functionality were observed in the IgG^+^IgM^+^/AI-low and IgG^+^IgM^+^/AI-high groups, IE1-specific T cells were rarely detected in the memory T cell pool during latent infection (IgG^+^IgM^−^ phenotype). Instead, pp65-specific T cells dominated the antiviral T cells in latently infected donors. This is similar to previous findings that antigens recognized by virus-specific CD8 T cells in MCMV-infected mice change with time since infection.^28^ However, whether the distinct fates of CMV-specific T cells depend solely on their targets remains unclear. A previous study demonstrated that high-affinity TCR-carrying T cells expand during primary MCMV infection but later decline due to cellular senescence; conversely, relatively low-affinity clones govern antiviral memory.^29^ In the present study, IE1-specific TCRs were restricted to the use of variable segments and were highly similar across donors, suggesting reproducible selection of IE1-specific clones in response to CMV infection. This limited TCR variation may prevent the emergence of IE1-specific clones capable of differentiating into long-lived memory T cells.

In our retrospective analysis of serological tests in a cohort of pregnant women, congenital infection was confirmed in 7.7% of pregnant women showing CMV IgM positivity and low AI within 16 weeks of gestation, which corresponded to the IgG^+^IgM^+^/AI-low group in the CD8 T cell analyses. This rate was lower than that reported in previous studies. In a previous review of clinical data presented in two independent studies in Belgium, 32% of pregnant women with low AI transmitted CMV to their neonates.^10^ Another study reported a 25% cCMV occurrence in IgM-positive donors with low/moderate AI in Italy.^12^ Ethnic and social backgrounds may affect the incidence of cCMV. Indeed, similar to this study, a large retrospective study conducted in Japan demonstrated a 7% incidence in a low AI population.^30^ Thus, the CMV immune response during pregnancy should be examined not only in Japanese but also in other ethnic groups. Another possible cause of this discrepancy is the limitations of the AI test. Although AI is widely accepted as a beneficial method for assessing the timing of primary infections, several issues have been identified in its interpretation. First, the low AI cut-off values vary depending on the testing method. Second, the length of time required to change from low to high AI states after primary infection largely varies among individuals. Given these considerations, the failure to differentiate between the CD8 profiles of the IgG^+^IgM^+^/AI-low and IgG^+^IgM^+^/AI-high groups in this study may also be attributed to the limitations of the AI test.

In this study, cases were categorized into IgG^+^IgM^+^/AI-low and IgG^+^IgM^+^/AI-high groups based on AI values. However, there were no clear distinctions in the properties of CMV-specific CD8 T cells between these groups. Furthermore, in the IgG^+^IgM^+^/AI-low and IgG^+^IgM^+^/AI-high groups, the characteristics of CMV-specific memory CD8 T cells were not completely consistent within the groups. For instance, in the IgG^+^IgM^+^/AI-low group, Tmp was predominant in P1-1 and P3-1, whereas GZMK^high^ Tem was predominant in P4; in the IgG^+^IgM^+^/AI-high group, Tmp was predominant in M1-1, M1-2, and M2, whereas GZMK^high^ Tem was predominant in M3 and P3-2. Additionally, although pp65-specific CD8 T cells were characteristic of past infections in the IgG^+^IgM^−^ group, this marker was present in nearly the same number in M2 in the IgG^+^IgM^+^/AI-high group as the IE1-specific CD8. Therefore, assessing the antigen specificity and subset composition of CMV-specific CD8 T cells may resolve potential issues in the conventional estimation of the timing of primary infection based on AI values, leading to more precise risk evaluations for cCMV.

MHC-dextramer scRNA-seq is highly costly and requires advanced technology for analysis. However, some of the distinct T cell profiles identified by MHC-dextramer scRNA-seq, were recapitulated by conventional flow cytometric analysis. In the IgG^+^IgM^−^ group, the level of pp65-specific CD8 T cells was higher than that of IE1-specific CD8 T cells. Additionally, the proportion of CMV-specific TEMRAs, identified as term-Tems in MHC-dextramer scRNA-seq, was higher in the IgG^+^IgM^−^ group than in the other groups. Furthermore, flow cytometry distinctly detected the expansion of IE1-specific T effectors in specimens from the IgG^+^IgM^+^/AI-low and IgG^+^IgM^+^/AI-high groups. These results suggested that insights gained from MHC-dextramer scRNA-seq could be translated into flow cytometry and other diagnostic tools in practice. In particular, a detailed evaluation of the humoral response to CMV has recently been promoted to distinguish between chronic and primary acute infection, and machine learning has been introduced to achieve high prediction accuracy.^31^ Considering the differences in T cell characteristics between acute and chronic infections, which were derived from this study, is expected to increase diagnostic accuracy.

In conclusion, antiviral CD8 T cell profiles associated with CMV serological patterns were indicative of latent and primary infections. In the primary and latent infection groups, distinctive features of the distribution of functional subsets and clonal composition were confirmed, corresponding to serological determinations. However, heterogeneity was observed in the profiles of CMV-specific T cells from donors with IgM persistence, especially where serological diagnosis is challenging. The findings of this study indicate that characterizing CD8 T cells is crucial for understanding the CMV infection phases in pregnant women.

### Limitations of the study

First, the number of MHC-dextramer scRNAseq cases in this study was limited, and we could not confirm statistically significant differences according to serological classification. However, some of the scRNA-seq findings, such as the predominance of effector memory CD8 T cells to pp65 in the IgG^+^IgM^−^ group and effector T cells to IE1 in the IgG^+^IgM^+^ group, can be statistically demonstrated using flow cytometry. Second, in this study, we isolated and characterized CMV-specific T cells using MHC dextramers and tetramers consisting of HLA-A∗24:02 with the immunodominant CMV epitopes from IE1 and pp65. However, the preference of target antigens by serological groups demonstrated in this study may not necessarily apply to other epitopes bound to the same or different HLA haplotypes. Third, the CMV activity of individual samples was not determined by virological aspects, such as CMV DNA detection using PCR, although this limitation is common to most studies on CMV-specific T cells in humans. Finally, our study did not address pregnancy-specific immunological changes, for which follow-up investigations after delivery are required.

## Resource availability

### Lead contact

Further information and requests for resources and reagents should be directed to and made available by the lead contact, Shuhei Sakakibara (sakakibara@ifrec.osaka-u.ac.jp).

### Materials availability

No unique materials were generated for this study.

### Data and code availability

• Data: Sequence data generated in this study have been deposited at the Japanese Genotype-phenotype Archive (JGA), which is hosted by the Bioinformation and DDBJ Center, under accession number JGAS000728, (JGA: JGAS000728). The data are available upon request if access is granted. To request access, contact National Bioscience Database Center (NBDC), Japan Science and Technology Agency, Department for Information Infrastructure.
• Code: The current study does not report original code(s).
• Additional information: Any additional information required to reanalyze the data reported in this paper is available from the lead contact upon reasonable request.

## Acknowledgments

We would like to thank Maki Kuronuma for technical support. We are also grateful to Daron Standley for generously providing a supportive research environment. Illustrations were created with BioRender.com. This study was supported by the Kanzawa Medical Research Foundation (A.T.) and JSPS Kakenhi (S.T. [no. 20K06610]).

## Author contributions

A.T., F.M., and S.T. contributed equally to this work; A.T., conceptualization, investigation, writing – original draft, and funding acquisition; F.M., data curation, investigation, and writing – original draft; S.T., formal analysis, writing – original draft, and funding acquisition; T.N., project administration and writing – review and editing; S.S, investigation and writing – original draft; T.O., data curation, investigation, and writing – review and editing; M.I., data curation and writing – review and editing; D.P., writing – review and editing; K.N., investigation and writing – review and editing; J.N., investigation and writing – review and editing; R.S., investigation and writing – review and editing; T.S., investigation and writing – review and editing; T.Ikeda., investigation and writing – review and editing; Y.N., investigation and writing – review and editing; T. Iriyama, data curation and writing – review and editing; D.O., investigation and writing – review and editing; H.O., writing – review and editing; J.B.W., writing – review and editing; Y.H., supervision and writing – review and editing; Y.O., supervision and writing – review and editing.

## Declaration of interests

The authors declare no competing interests.

## STAR★Methods

### Key resources table

**Table:** 

REAGENT or RESOURCE	SOURCE	IDENTIFIER
**Antibodies**		

Dextramer(10x)-Gold, HLA-A∗2402/AYAQKIFKI/PE	Immudex	Cat#WF02196DXG PE
Dextramer(10x)-Gold, HLA-A∗2402/QYDPVAALF/PE	Immudex	Cat#WF02133DXG PE
Dextramer (10x)-Gold, HLA-A∗2402/VYALPLKML/PE	Immudex	Cat#WF02134DXG PE
Dextramer (10x)-Gold, HLA-A∗0201/ALIAPVHAV	Immudex	Cat#WB02666
T-Select HLA-A∗24:02 CMV pp65 Tetramer-QYDPVAALF-PE	MBL Life Science	Cat#TS-0020-1C
HLA A2402_CMV IE1 (AYAQKIFKI)-APC	ImmunoAware	Cat#1020-03-50
Anti-CD8a-V421	BD Biosciences	Clone Hit8a; Cat#740078, PRID: N/A
Anti-CD19-PerCPCy5.5	BioLegend	Clone HIB19; Cat#302230; RRID: AB_2073119
Anti-CD66b-PerCPCy5.5	BioLegend	Clone G10F5; Cat#305108; PRID: AB_2077856
Anti-CD14-PerCPCy5.5	BioLegend	Clone 63D3; Cat#367109; RRID: AB_2566711
Anti-CCR7-PECy7	BioLegend	Clone G043H7; Cat#353226; RRID: AB_11126145
Anti-CD45RA-APCCy7	BioLegend	Clone HI100; Cat#304128; RRID: AB_10708880
Anti-CD127-FITC	BioLegend	Clone A019D5; Cat#351312; RRID: AB_10897643

**Biological samples**		

Human whole blood	The University of Tokyo Hospital	N/A

**Chemicals, peptides, and recombinant proteins**		

7-Aminoactinomycin D (7AAD)	BioLegend	Cat#420404
Pierce™ Universal nuclease for cell lysis	Thermo Scientific	Cat#88702
Bovine serum albumin, fatty-acid free, low endotoxin	Sigma-Aldrich	Cat#A8806
Bovine serum albumin, globulin-free	Nacalai Tesque	Cat#01281-26
D-PBS(−)	Fujifilm Wako	Cat#043-29791
Cell Banker 1	Takara/Zenogen Pharma	Cat#CB011
Pierce™ 16% Formaldehyde (w/v), Methanol-free	Thermo Scientific	Cat#28906

**Critical commercial assays**		

QuickGene DNA whole blood kit S	Kurabo	Cat#636-23541
WAKFlow HLA Typing reagent for HLA-A	Wakunage	Cat#4N705
Vacutainer CPT mononuclear cell preparation tube - sodium heparin	BD Biosciences	Cat#362753
Seiken CMV IgM EIA	Denka Seiken	Cat#325754
Seiken CMV IgG EIA	Denka Seiken	Cat#322647
Enzygnost anti-CMV kit	Siemens Healthineers	N/A
Architect CMV IgG avidity assay kit	Abbott	Cat#3L46
Chromium Next GEM Chip G single cell kit	10x Genomics	Cat#1000127
Chromium Next GEM single cell 5ʹ library and gel bead kit v2	10x Genomics	Cat#1000263
Chromium single cell V(D)J enrichment kits, human T cell	10x Genomics	Cat#1000005

**Deposited data**		

MHC dextramer scRNA-seq data	This study	JGA: JGAS000728

**Software and algorithms**		

FlowJo, ver 10.9	Treestar	N/A
EZR, ver 1.55	Kanda^32^	https://www.jichi.ac.jp/saitama-sct/SaitamaHP.files/statmedEN.html
R 4.3.1	The R Foundation	https://www.r-project.org/
cellranger 6.1.2	10x Genomics	https://www.10xgenomics.com/
Scanpy 1.9.3	Wolf et al.^33^	https://pypi.org/project/scanpy/
Scirpy 0.13.0	Sturm et al.^34^	https://pypi.org/project/scirpy/

### Experimental model and study participant details

#### Ethics

All experimental procedures were approved by the Institutional Review Board of the University of Tokyo (approval number: 12052) and were conducted in accordance with the Ethical Guidelines for Medical and Health Research Involving Human Subjects issued by the Ministry of Education, Culture, Sports, Science and Technology, the Ministry of Health, Labor and Welfare, Japan in line with the WMA’s Declaration of Helsinki. Written informed consent was obtained from all patients.

#### Clinical participants

For serological analysis, the clinical information of 8,552 pregnant women who delivered at the University of Tokyo Hospital between January 2013 and December 2020 was retrospectively investigated. Among these, CMV antibody titers and background data were investigated for 3,122 pregnancies with available CMV antibody data up to 16 weeks of gestation (Table S1). Pregnant women who were CMV IgG negative up to 16 weeks of gestation were diagnosed as uninfected and educated on infection prevention. CMV IgG- and IgM-positive donors were further tested for CMV AI. For the 240 pregnant women in this cohort, CMV serology was tested in two pregnancies (P1: first pregnancy; P2: subsequent pregnancy). A demographic summary of the donors is presented in Table S2.

For MHC dextramer scRNA-seq and flow cytometry analyses, blood samples were collected from HLA-A∗24:02 pregnant women who visited the University of Tokyo Hospital between February 2019 and June 2021 (Table 1 and S7). These specimens were classified into the following four groups: IgG^+^IgM^+^/AI-low group, CMV IgG-positive, IgM-positive with low IgG avidity; IgG^+^IgM^+^/AI-high group, IgG-positive, IgM-positive with high IgG avidity, including specimens from donors exhibiting IgG^+^IgM^+^/AI-low pattern earlier and IgG AI increased from low during pregnancy; IgG^+^IgM^−^ group, CMV IgG-positive and CMV IgM-negative; and IgG^−^ group, CMV IgG-negative. In selecting donors, we focused on IgM-positive cases to characterize CD8 T cells, especially in the primary infection and persistent IgM groups. IgG+IgM-cases were selected as a comparative group. In addition, the analysis of samples for which time-series samples were available and AI had changed throughout the gestational period was prioritized to elucidate the changes in T cells due to changes in AI.

### Method details

#### HLA typing

DNA was extracted from whole blood samples using the QuickGene DNA Whole Blood Kit S (Kurabo, Osaka, Japan). HLA typing was performed using the WAKFlow HLA Typing Kit (Wakunaga) in accordance with the manufacturer’s instructions.

#### CMV serological testing

The reagents used in the assay are listed in the key resources table of STAR Methods. CMV IgG and IgM antibody titers were measured using the enzyme immunoassay (EIA) method with the "Seiken" viral antibody kit from Denka Seiken Co. in laboratories of SRI, a testing company certified in accordance with international standards. A plate coated with anti-human IgM (or IgG) antibody (mouse) was added with the antibody solution, incubated at 20°C–30°C for 1 h, washed twice, added with the CMV antigen solution, incubated at 20°C–30°C for 1 h, washed twice, added with the enzyme-labeled CMV monoclonal antibody, incubated at 20°C–30°C for 1 h, washed four times, added with the substrate solution, incubated at 20°C–30°C for 30 min, and then added with the stop solution. Serum levels were measured using an auto reader (main wavelength 450 nm/sub wavelength 600–700 nm) with a blank well as control within 30 min. The cut-off values for CMV IgG and IgM were set at 2.0 and 0.8, respectively. The CMV IgG AI was measured using the Enzygnost anti-CMV kit (Siemens) and the Architect CMV IgG Avidity kit (Abbott).^35^^,^^36^ Low AI was defined as an AI of <40% for the Siemens Healthcare kit and <50% for the Abbott kit.

#### MHC-dextramer scRNA-seq

Antibodies, dextramers, and other reagents used in MHC-dextramer scRNA-seq are listed in the key resources table of STAR Methods. Blood samples were collected from pregnant women with varying CMV infection phases, as determined by CMV serological test results during their early pregnancies. The breakdown is as follows: CMV IgG^+^IgM^+^/low AI (*n* = 7, *N* = 4); CMV IgG^+^IgM^+^/high AI (*n* = 4, *N* = 3); CMV IgG^+^IgM^−^ (*n* = 3, *N* = 2); and CMV IgG^−^IgM^-^ (*n* = 1, *N* = 1) (Table 1). PBMCs were isolated from blood samples using a Vacutainer CPT Mononuclear Cell Preparation Tube in accordance with the manufacturer’s protocol and cryopreserved in a Cellbanker (Takara) until use. CMV-specific CD8 T cells were labeled with 10X Genomics-compatible, PE-conjugated dCODE dextramer (Immudex), and fluorophore-conjugated antibodies (key resources table of STAR Methods) in accordance with the manufacturer’s instructions. 7AAD^−^ CD4^−^ CD14^−^ CD19^−^ CD66b^−^ CD8a^+^ dextramer^+^ cells were sorted using a FACS Aria III (BD Biosciences). For gating, PBMCs from uninfected donors (IgG^−^ group) were used as negative controls. The sorted cells were captured in PBS containing 0.1% bovine serum albumin (BSA, fatty-acid free [Sigma-Aldrich]) and subjected to scRNA-seq using the Chromium Single Cell 5′ Library (10X Genomics). Single-cell RNA libraries and TCR libraries were prepared using the Chromium Single Cell 5′ Kit v2 and Chromium Single Cell V(D)J Kit, respectively (10X Genomics), in accordance with the manufacturer’s instructions. These libraries were sequenced on a NovaSeq 6000 (Illumina) at a read length of 28 × 90 to yield a minimum of 20,000 reads per cell for gene expression and 5000 reads per cell for VDJ and feature barcoding.

#### Single-cell data processing

The FASTQ files were processed by cellranger v6.1.2 (10x Genomics) with the “multi” option, where refdata-gex-GRCh38-2020-A and refdata-cellranger-vdj-GRCh38-alts-ensembl-5.0.0 were specified for the reference for gene expression and TCRs, respectively. The Python package Scanpy v1.9.3^33^ was used for downstream scRNA-seq data analysis. TCR sequences were processed for genotyping using Scirpy v0.13.0.^34^ To avoid the influence of immune repertoire distribution on multivariate gene expression analysis, we excluded TCR fragment genes from the gene expression data. For the quality control of single cells, we first removed cells that expressed less than 200 genes. Then, we retained cells whose gene expression levels were not extreme; specifically, the cells whose number of expressed genes was less than 1000 or greater than 4000 and the cells in which the total counts of gene expression were less than 2000 or greater than 10000 were removed to avoid dead cells or multiplets. We removed cells with a higher percentage of mitochondrial gene count (greater than 10%). This preprocessing reduced the number of cells from 23,446 to 13,710. Among them, 10,560 cells were detected using TCR sequence data. After undergoing these quality control processes, data from the two runs were merged using the combat function of Scanpy, which implements the ComBat algorithm for batch effect correction.^37^ The donors for each cell type were identified using hashtag counts. The minimum count for each hashtag required for donor identification was manually determined from a histogram of distributions. The epitope specificity of each TCR was similarly evaluated using dextramer counts. Dimensionality reduction was performed using the *umap* function of Scanpy, followed after PCA. Leiden clustering was performed using the *leiden* function with a resolution of 0.3. After UMAP visualization and clustering, actual analysis was performed for cells with unique identification to a single donor and epitope specificity to avoid ambiguity. The number of cells specific to ALIAPVHAV (control) and VYALPLKML (CMV pp65) dextramers was only one for each; therefore, we removed these cells. L1-2 and N1 were excluded from the analysis because only one cell was identified in each. The smallest cluster in the Leiden clustering was abundant with doublet cells, had only one cell after those prescriptions, and was excluded from further analysis. As a result, we obtained 9057 cells for downstream analysis. For clonotype analysis, we further restricted the cells to those with a single pair of TCR alpha and beta chains.

#### Flow cytometry

Antibodies and other reagents used in the assay are listed in the key resources table of STAR Methods. A single suspension of cells was incubated with tetramer reagents in PBS containing 1% BSA (Nacalai Tesque) for 30 min and further stained with the fluorophore-conjugated antibodies listed in the key resources table for 15 min. Washed cells were suspended in PBS containing 7AAD (BioLegend), fixed with 2% formaldehyde, and analyzed using FACS Aria III (BD). Data analysis was performed using FlowJo v10.9 (Treestar). More than 5000 CD8 T cells were analyzed (IgG^+^IgM^+^/AI-low group, *n* = 7; IgG^+^IgM^+^/AI-high group, *n* = 10; and IgG^+^IgM^−^ group, *n* = 19). Samples with more than five tetramer-positive CD8 T cells were used for the phenotypical analysis (IgG^+^IgM^+^/AI-low group, *n* = 6; IgG^+^IgM^+^/AI-high group, *n* = 8; and IgG^+^IgM^−^ group *n* = 18). CCR7^−^CD45RA^−^, CCR7^−^CD45RA^+^IL7R^−^, and CCR7^−^CD45RA^+^IL7R^+^ were defined as effector memory, effector, and TEMRA, respectively.^25^

### Quantification and statistical analysis

All statistical analyses were conducted using EZR (Version 1.55).^32^ A paired t test was used to analyze the CMV IgM titers. For flow cytometric analysis, the Kruskal–Wallis test, post-hoc test with Holm correction, and two-way ANOVA were used. Statistical significance was set at *p* < 0.05.

## Supplemental information

Document S1. Figures S1–S7

Table S1. Clinical summary of pregnant donors for serological data

Table S2. Clinical summary of donors with serological data of two pregnancies

Table S3. Number of tetramer-positive cells used in the scRNAseq analysis

Table S4. Summary of top 10 clones in each sample

Table S5. Summary of cell-based TCR fragment

Table S6. Clonotype sharing

Table S7. Clinical summary of donors for flow cytometry

## References

[1] Cannon M.J., Schmid D.S., Hyde T.B. (2010). Review of Cytomegalovirus Seroprevalence and Demographic Characteristics Associated with Infection. Rev. Med. Virol..

[2] Drew W.L. (1992). Cytomegalovirus Infection in Patients with AIDS. Clin. Infect. Dis..

[3] Kotton C.N. (2013). CMV: Prevention, Diagnosis and Therapy. Am. J. Transplant..

[4] Rawlinson W.D., Boppana S.B., Fowler K.B., Kimberlin D.W., Lazzarotto T., Alain S., Daly K., Doutré S., Gibson L., Giles M.L. (2017). Congenital Cytomegalovirus Infection in Pregnancy and the Neonate: Consensus Recommendations for Prevention, Diagnosis, and Therapy. Lancet Infect. Dis..

[5] Fowler K.B., Boppana S.B. (2006). Congenital Cytomegalovirus (CMV) Infection and Hearing Deficit. J. Clin. Virol..

[6] Ross S.A., Boppana S.B. (2005). Congenital Cytomegalovirus Infection: Outcome and Diagnosis. Semin. Pediatr. Infect. Dis..

[7] Malm G., Engman M.L. (2007). Congenital Cytomegalovirus Infections. Semin. Fetal Neonatal Med..

[8] Friedman S., Ford-Jones E.L. (1999). Congenital Cytomegalovirus Infection – an Update. Paediatr. Child Health.

[9] Coll O., Benoist G., Ville Y., Weisman L.E., Botet F., Anceschi M.M., Greenough A., Gibbs R.S., Carbonell-Estrany X., WAPM Perinatal Infections Working Group (2009). Guidelines on CMV Congenital Infection. J. Perinat. Med..

[10] Prince H.E., Lapé-Nixon M. (2014). Role of Cytomegalovirus (CMV) IgG Avidity Testing in Diagnosing Primary CMV Infection During Pregnancy. Clin. Vaccine Immunol..

[11] Lilleri D., Gerna G., Furione M., Zavattoni M., Spinillo A. (2016). Neutralizing and ELISA IgG Antibodies to Human Cytomegalovirus Glycoprotein Complexes May Help Date the Onset of Primary Infection in Pregnancy. J. Clin. Virol..

[12] Lazzarotto T., Guerra B., Gabrielli L., Lanari M., Landini M.P. (2011). Update on the Prevention, Diagnosis and Management of Cytomegalovirus Infection During Pregnancy. Clin. Microbiol. Infect..

[13] Sellier Y., Guilleminot T., Ville Y., Leruez-Ville M. (2015). Comparison of the LIAISON(®) CMV IgG Avidity II and the VIDAS(®) CMV IgG Avidity II Assays for the Diagnosis of Primary Infection in Pregnant Women. J. Clin. Virol..

[14] Iijima S. (2022). Pitfalls in the Serological Evaluation of Maternal Cytomegalovirus Infection as a Potential Cause of Fetal and Neonatal Involvements: A Narrative Literature Review. J. Clin. Med..

[15] Chen D.G., Xie J., Su Y., Heath J.R. (2023). T Cell Receptor Sequences Are the Dominant Factor Contributing to the Phenotype of CD8^+^ T Cells with Specificities Against Immunogenic Viral Antigens. Cell Rep..

[16] Erickson J.R., Stevens-Ayers T., Mair F., Edmison B., Boeckh M., Bradley P., Prlic M. (2022). Convergent Clonal Selection of Donor- and Recipient-Derived CMV-Specific T Cells in Hematopoietic Stem Cell Transplant Patients. Proc. Natl. Acad. Sci. USA.

[17] Nakaoka H., Inoue I. (2015). Distribution of HLA Haplotypes Across Japanese Archipelago: Similarity, Difference and Admixture. J. Hum. Genet..

[18] Remmerswaal E.B.M., Hombrink P., Nota B., Pircher H., Ten Berge I.J.M., van Lier R.A.W., van Aalderen M.C. (2019). Expression of IL-7Ralpha and KLRG1 Defines Functionally Distinct CD8^+^ T-Cell Populations in Humans. Eur. J. Immunol..

[19] Kanbar J.N., Ma S., Kim E.S., Kurd N.S., Tsai M.S., Tysl T., Widjaja C.E., Limary A.E., Yee B., He Z. (2022). The Long Noncoding RNA Malat1 Regulates CD8+ T Cell Differentiation by Mediating Epigenetic Repression. J. Exp. Med..

[20] Notarbartolo S., Ranzani V., Bandera A., Gruarin P., Bevilacqua V., Putignano A.R., Gobbini A., Galeota E., Manara C., Bombaci M. (2021). Integrated Longitudinal Immunophenotypic, Transcriptional and Repertoire Analyses Delineate Immune Responses in COVID-19 Patients. Sci. Immunol..

[21] Bourque J., Kousnetsov R., Hawiger D. (2022). Roles of Hopx in the Differentiation and Functions of Immune Cells. Eur. J. Cell Biol..

[22] Kitaura K., Shini T., Matsutani T., Suzuki R. (2016). A New High-Throughput Sequencing Method for Determining Diversity and Similarity of T Cell Receptor (TCR) Alpha and Beta Repertoires and Identifying Potential New Invariant TCR Alpha Chains. BMC Immunol..

[23] Toya T., Taguchi A., Kitaura K., Misumi F., Nakajima Y., Otsuka Y., Konuma R., Adachi H., Wada A., Kishida Y. (2020). T-Cell Receptor Repertoire of Cytomegalovirus-Specific Cytotoxic T-Cells After Allogeneic Stem Cell Transplantation. Sci. Rep..

[24] Klenerman P., Oxenius A. (2016). T Cell Responses to Cytomegalovirus. Nat. Rev. Immunol..

[25] Harari A., Bellutti Enders F., Cellerai C., Bart P.A., Pantaleo G. (2009). Distinct Profiles of Cytotoxic Granules in Memory CD8 T Cells Correlate with Function, Differentiation Stage, and Antigen Exposure. J. Virol..

[26] Minervina A.A., Pogorelyy M.V., Kirk A.M., Crawford J.C., Allen E.K., Chou C.H., Mettelman R.C., Allison K.J., Lin C.Y., Brice D.C. (2022). SARS-CoV-2 Antigen Exposure History Shapes Phenotypes and Specificity of Memory CD8^+^ T Cells. Nat. Immunol..

[27] Munks M.W., Rott K., Nesterenko P.A., Smart S.M., Williams V., Tatum A., Xu G., Smith T., Murray S.E., Hill A.B. (2023). Latent CMV Infection of Lymphatic Endothelial Cells Is Sufficient to Drive CD8 T Cell Memory Inflation. PLoS Pathog..

[28] Munks M.W., Gold M.C., Zajac A.L., Doom C.M., Morello C.S., Spector D.H., Hill A.B. (2006). Genome-Wide Analysis Reveals a Highly Diverse CD8 T Cell Response to Murine Cytomegalovirus. J. Immunol..

[29] Schober D.J., Hunt B.R., Benjamins M.R., Saiyed N.S., Silva A., De Maio F.G., Homan S.M. (2021). Homicide Mortality Inequities in the 30 Biggest Cities in the U.S. Am. J. Prev. Med..

[30] Shimada K., Toriyabe K., Kitamura A., Morikawa F., Minematsu T., Ikejiri M., Suga S., Toyoda H., Amano K., Kitano M. (2021). Primary Cytomegalovirus Infection During Pregnancy and Congenital Infection: a Population-Based, Mother–Child, Prospective Cohort Study. J. Perinatol..

[31] Hederman A.P., Remmel C.A., Sharma S., Natarajan H., Weiner J.A., Wrapp D., Donner C., Delforge M.L., d'Angelo P., Furione M. (2024). Discrimination of Primary and Chronic Cytomegalovirus Infection Based on Humoral Immune Profiles in Pregnancy. J. Clin. Investig..

[32] Kanda Y. (2013). Investigation of the Freely Available Easy-to-Use Software “EZR” for Medical Statistics. Bone Marrow Transplant..

[33] Wolf F.A., Angerer P., Theis F.J. (2018). SCANPY: Large-Scale Single-Cell Gene Expression Data Analysis. Genome Biol..

[34] Sturm G., Szabo T., Fotakis G., Haider M., Rieder D., Trajanoski Z., Finotello F. (2020). Scirpy: a Scanpy Extension for Analyzing Single-Cell T-Cell Receptor-Sequencing Data. Bioinformatics.

[35] Curdt I., Praast G., Sickinger E., Schultess J., Herold I., Braun H.B., Bernhardt S., Maine G.T., Smith D.D., Hsu S. (2009). Development of Fully Automated Determination of Marker-Specific Immunoglobulin G (IgG) Avidity Based on the Avidity Competition Assay Format: Application for Abbott Architect Cytomegalovirus and Toxo IgG Avidity Assays. J. Clin. Microbiol..

[36] Ebina Y., Minematsu T., Morioka I., Deguchi M., Tairaku S., Tanimura K., Sonoyama A., Nagamata S., Morizane M., Yamada H. (2015). Rapid Increase in the Serum Cytomegalovirus IgG Avidity Index in Women with a Congenitally Infected Fetus. J. Clin. Virol..

[37] Johnson W.E., Li C., Rabinovic A. (2007). Adjusting Batch Effects in Microarray Expression Data Using Empirical Bayes Methods. Biostatistics.

