# Supplementary material for: Multifaceted profiling of virus-specific CD8 T cells reveals distinct immune signatures against cytomegalovirus infection states during pregnancy
Source: iScience. 2025 Apr 11;28(5):112416. doi: 10.1016/j.isci.2025.112416 (PMC12059710; doi:10.1016/j.isci.2025.112416)
Supplement: Document S1. Figures S1–S7 [file mmc1.pdf]

iScience, Volume *28*

Supplemental information

Multifaceted profiling of virus-specific CD8 T cells reveals distinct immune signatures against cytomegalovirus infection states during pregnancy

Ayumi Taguchi, Fumi Misumi, Shunsuke Teraguchi, Takeshi Nagamatsu, Shuhei Sakakibara, Tomohiro Otani, Mari Ichinose, David Priest, Kazuki Nakajima, Junko Nakamura, Ryoko Sawada, Tatsuo Suzutani, Toshiyuki Ikeda, Yutaka Nagura, Takayuki Iriyama, Daisuke Okuzaki, Hitoshi Okazaki, James B. Wing, Yasushi Hirota, and Yutaka Osuga

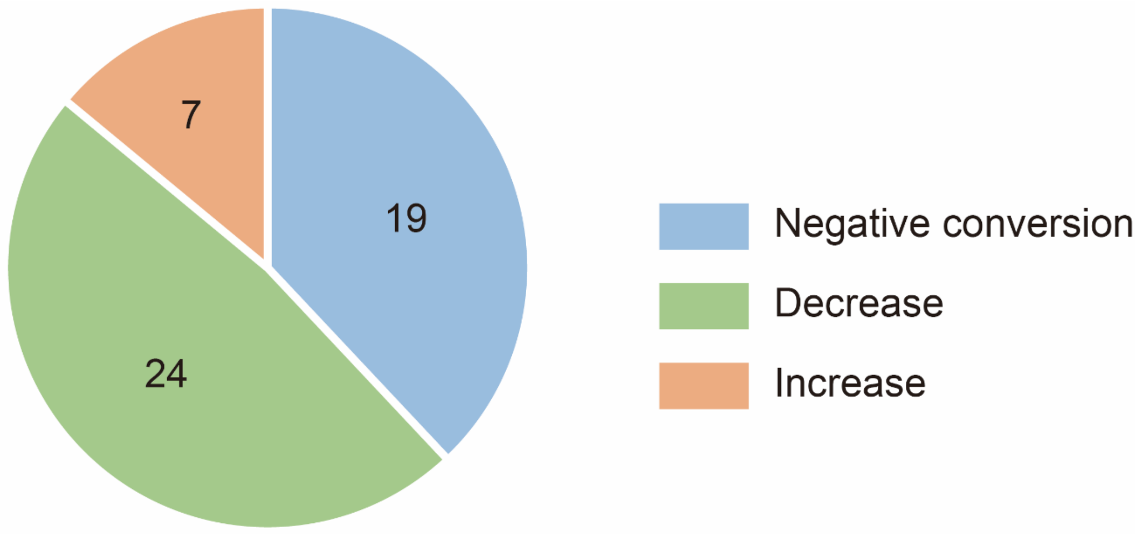

Figure S1. Longitudinal analysis of CMV IgM titers. Early (<16 weeks) CMV IgG and IgM-positive donors underwent multiple test results with an interval of more than 100 days between the initial and subsequent tests or confirmed negative conversion of IgM during pregnancy. N = 50.

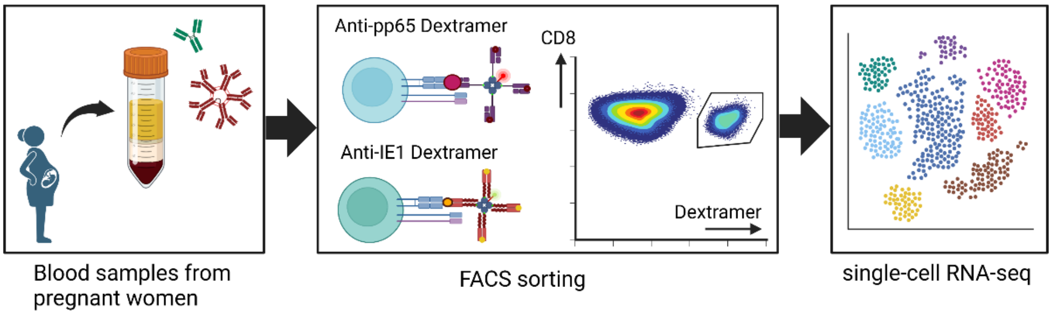

Figure S2. Schematic presentation of single-cell transcriptome analysis for CMV-specific CD8 T cells. Blood samples were collected from pregnant women with different anti-CMV serologic test results. For some donors, blood was collected at two different time points during pregnancy. PBMCs were incubated with PE-conjugated HLA24*02-CMV pp65 and IE1 dextramers and sample-specific Hashtag. Dextramer-bound CD8+ cells were sorted and subjected to Chromium 10x droplet scRNAseq.

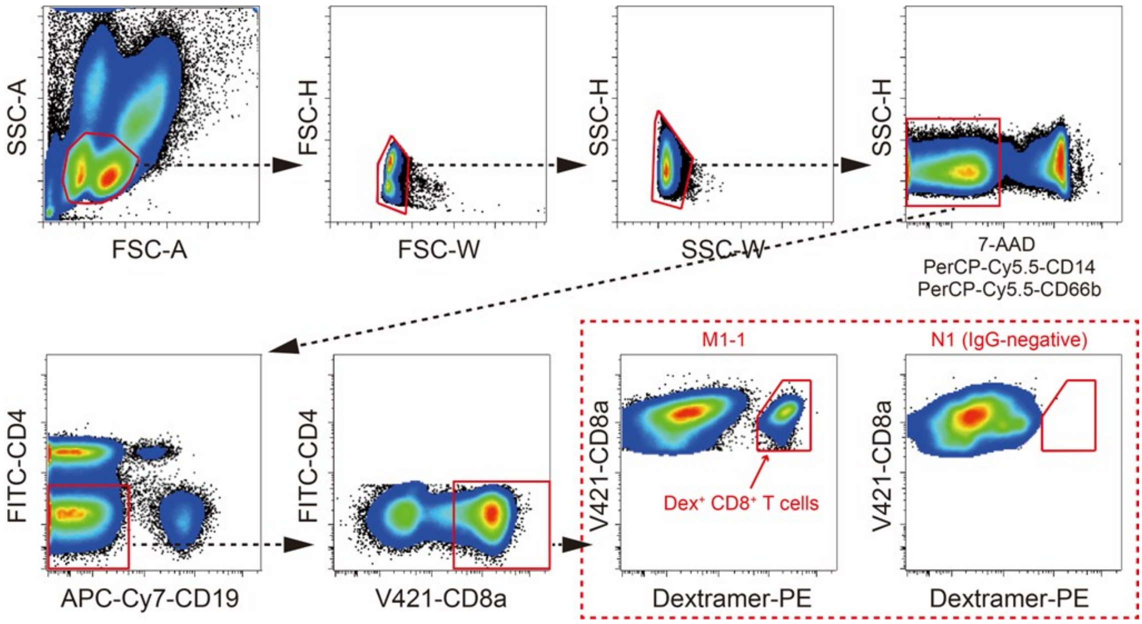

Figure S3. Gating strategy for single cell RNA sequence. Representative gating strategies were shown; 7AAD- CD4- CD14- CD19- CD66b- CD8a+ dextramer+ cells were sorted.

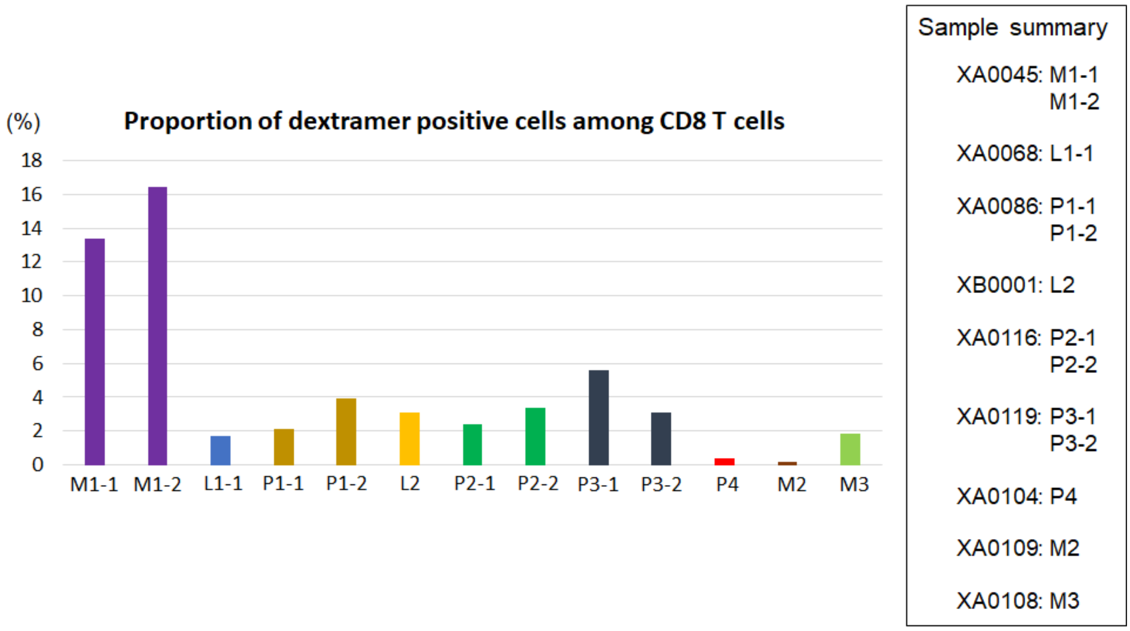

Figure S4. The percentage of dextramer-positive cells varies by donors. The proportion of dextramer-positive cells were determined by flow cytometry. The bars are colored by donors.

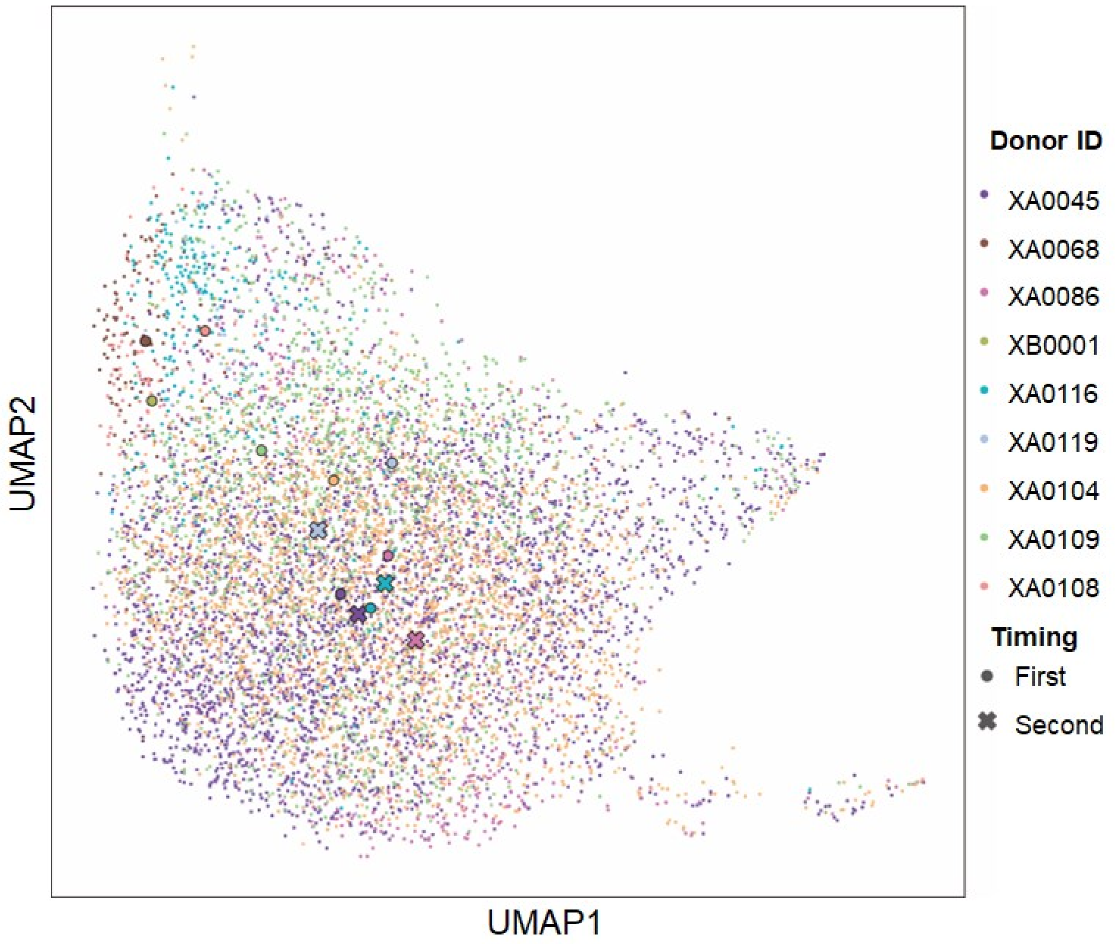

Figure S5. Distribution of CMV-specific CD8 T cells by samples. Distribution of T cells by sample were projected on the UMAP. The UMAP plots (● and ×) depicts the pseudo-bulk profiles of CMV-specific CD8 T cells from each sample.

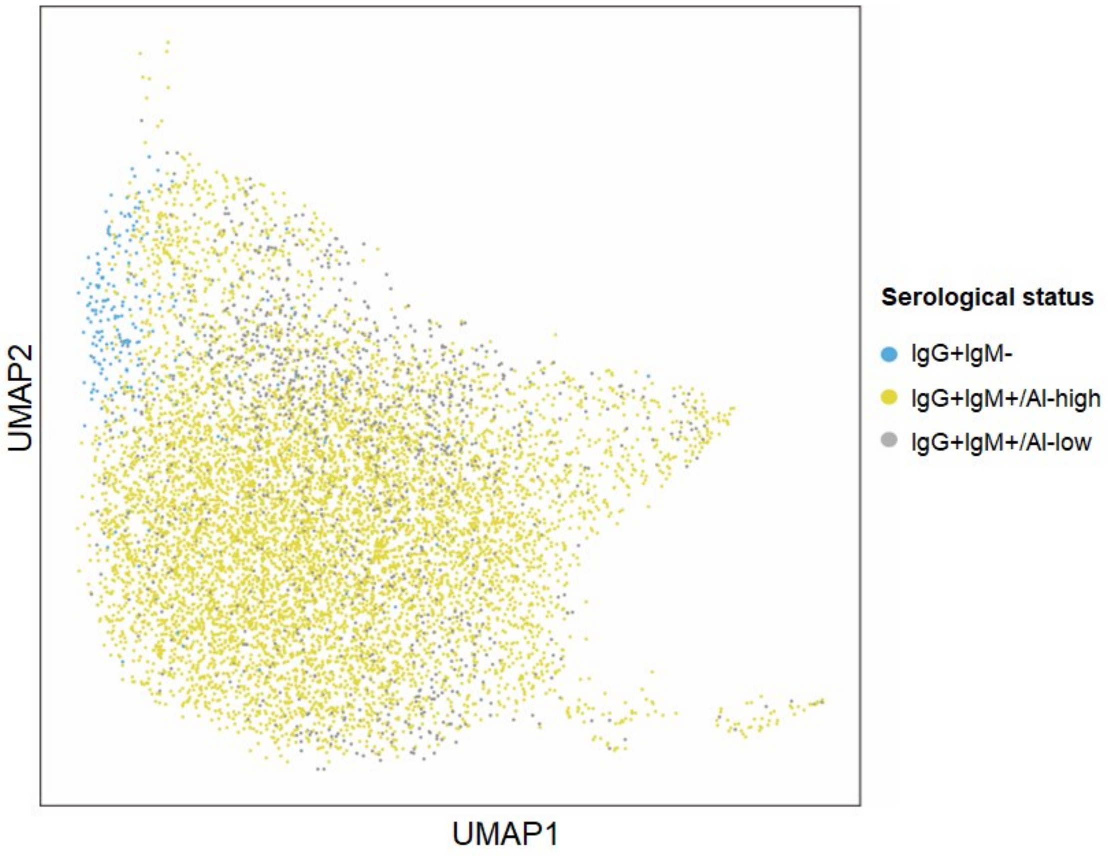

Figure S6. Distribution of CMV-specific CD8 T cells by serological status. Distribution of T cells by serological status were projected on the UMAP.

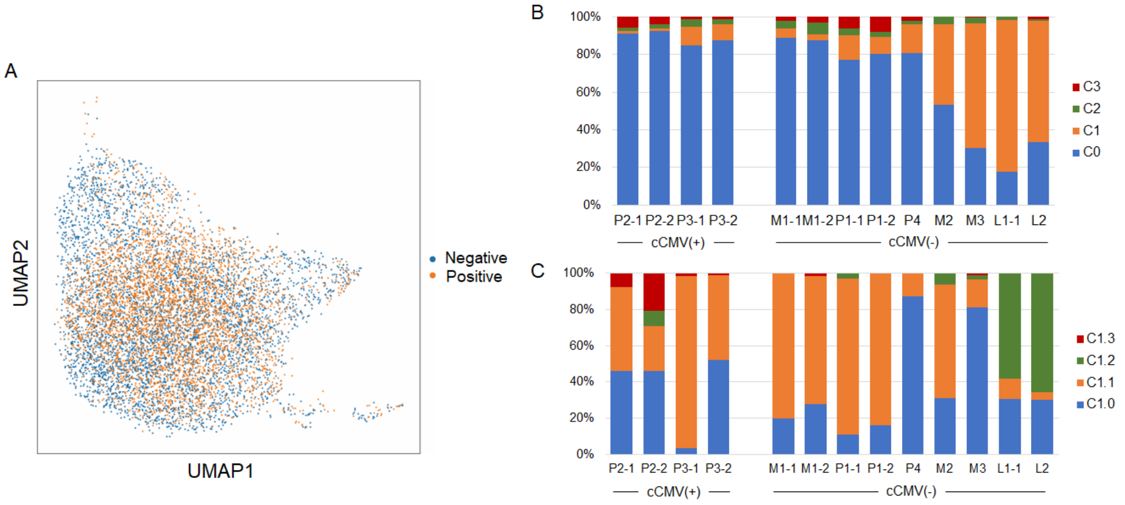

Figure S7. Profiles of CMV-specific CD8 T cells according to the presence of congenital CMV (cCMV) infection. (A) Distribution of CMV-specific CD8 T cells by cCMV-positivity. (B) Proportion of T cell subsets in individual samples according to cCMV-positivity. (C) Proportion of memory T cell subsets in individual samples according to cCMV-positivity.
